# Bacterial Nanocellulose from Side-Streams of Kombucha Beverages Production: Preparation and Physical-Chemical Properties

**DOI:** 10.3390/polym9080374

**Published:** 2017-08-18

**Authors:** Stefan-Ovidiu Dima, Denis-Mihaela Panaitescu, Csongor Orban, Marius Ghiurea, Sanda-Maria Doncea, Radu Claudiu Fierascu, Cristina Lavinia Nistor, Elvira Alexandrescu, Cristian-Andi Nicolae, Bogdan Trică, Angela Moraru, Florin Oancea

**Affiliations:** 1 INCDCP ICECHIM, 202 Splaiul Independentei, Bucharest 060021, Romania; phd.Ovidiu.Dima@gmail.com (S.-O.D.); Panaitescu@icf.ro (D.-M.P.); Ghiurea@gmail.com (M.G.); SandaMariaDoncea@gmail.com (S.-M.D.); Radu_Claudiu_Fierascu@yahoo.com (R.C.F.); LC_Nistor@yahoo.com (C.L.N.); ElviraAlexandrescu@yahoo.com (E.A.); CA_Nicolae@yahoo.com (C.-A.N.); Trica.Bogdan@gmail.com (B.T.); 2 S.C. Corax Bioner CEU S.A., 53 Sarkadi Elek, Miercurea Ciuc 530200, Romania; csopont@gmail.com; 3 S.C. Laboratoarele Medica Srl, 11 Frasinului Str., Otopeni 075100, Romania; Angela.Moraru@medicagroup.ro

**Keywords:** nanocellulose, Kombucha membranes, spray-drying, microfluidization, bacterial nanofibrils

## Abstract

We focused on preparing cellulose nanofibrils by purification, separation, and mechanical treatment of Kombucha membranes (KM) resulted as secondary product from beverage production by fermentation of tea broth with symbiotic culture of bacteria and yeast (SCOBY). We purified KM using two alkaline solutions, 1 and 4 M NaOH, which afterwards were subjected to various mechanical treatments. Transmission electron microscopy (TEM), scanning electron microscopy (SEM), dynamic light scattering (DLS), X-ray diffraction (XRD), X-ray fluorescence (XRF), Fourier-transform infrared spectroscopy (FTIR), and thermogravimetric analysis (TGA) were employed to evaluate the purification degree, the size and aspect of cellulose fibrils after each treatment step, the physical-chemical properties of intermediary and final product, and for comparison with micro-crystalline cellulose from wooden sources. We determined that 1 M NaOH solution leads to approx. 85% purification, while a higher concentration assures almost 97% impurities removal. XRD analysis evidenced an increase in crystallinity from 37% to 87% after purification, the characteristic diffractograms of Iα and Iβ cellulose allomorphs, and a further decrease in crystallinity to 46% after microfluidization, fact correlated with a drastically decrease in fibrils’ size. FTIR analysis evidenced the appearance of new chain ends by specific transmission bands at 2941 and 2843 cm^−1^.

## 1. Introduction

Nanocellulose (NC) is one of the most intensively studied biopolymers at the moment due to its appealing properties like high mechanical strength, high surface area, chemical stability, hydrophilicity, crystallinity, transparency, biocompatibility, magnetic and electric susceptibility, proton conductivity, rich surface chemistry, and availability, cellulose being the most abundant renewable organic material produced in the biosphere [1,2,3,4]. These properties make NC very interesting for various applications in multiple domains. In the biomedical and pharmaceutical fields NC can be used for wound dressing, virus-removal filters, drug-delivery systems, scaffolds, tissue regeneration, or bioprinting [1,5,6,7,8,9,10]. In food industry, NC can be used as functional food ingredient, food stabilizer, or as nanofiller in nanocomposites with gas-barrier properties for food packaging [1,11,12,13,14]. NC was used also in aerogels, hydrogels, membranes, and other types of adsorbents for wastewater treatment and advanced separation processes due to its hydrophilicity, biodegradability, and adsorption properties [15,16,17,18,19,20]. NC found applications even in the development of high tech energy devices, triboelectric nanogenerators, flexible transistors, (bio)sensors, and other devices for nanotechnology, energy storage, and photonics [1,3,21,22,23,24,25,26].

Although cellulose is the most abundant biopolymer on Earth, it is a hard task to obtain nanocellulose from the complex vegetal matrices in which cellulose participates to form a though, intercalated network with other biopolymers like lignin, pectin, and hemicellulose. Strong acids, alkali, or particular solvents are required to break down this complex vegetal matrix, which makes these methods inconvenient from environmental and economic reasons.

An alternative to NC from biomass is the production of NC through microbial processes, the resulting NC being also known as bacterial nanocellulose (BNC). The advantages of BNC are the high cellulose purity compared to plant sources, higher flexibility, higher hydrophilicity, and drug load-release properties [22,27,28]. The disadvantages are the long production time (up to 21 days), the need of axenic conditions for growing specific bacteria, and the reaction conditions of the biosynthesis.

A convenient BNC source could be the Kombucha membrane (KM), a by-product of the wellness beverages industry. KMs are cellulosic pellicles obtained during the fermentation of black or green tea broth, with 5–8% added sucrose, using a symbiotic consortium of bacteria and yeast (SCOBY). The main purpose of the process is the production of a slightly alcoholic (0.5–2.5%) refreshment drink called Kombucha, or of a vinegar, when fermentation is prolonged. This kind of fermented tea has ancient origins; according to some sources it originated in Manchuria, northeast China, around 220 B.C., when it was appreciated for its detoxifying and energizing properties [29]. Nowadays, nutritionists and dietitians, accompanied by researchers, argue that Kombucha beverage detoxifies the body [30], quells pain, fortifies the immune system [31], treats gastric ulcers [32], and gives an energy boost. Other studies are evidencing its hypoglycemic and antilipidemic properties [33], as well as its antimicrobial, antioxidant, and even anti-carcinogenic properties [29,31,34].

The liquid phase of the fermentation process, or the “soup”, contains high amounts of antioxidants, phenols and polyphenols, glucuronic acid, d-saccharic acid-1,4-lactone, and B-vitamin complex [31]. Its high content in probiotics (beneficial gut bacteria, e.g., lactobacilli) is important for human health, since probiotics are also called “the forgotten organ” or “the neglected endocrine organ” [32,35]. The solid phase that is formed at the soup-air interface in 3 to 21 days of fermentation is a cellulose-based pellicle known as tea fungus. This microbial biofilm consists in a multi-layered matrix of entangled bacterial cellulose nano and microfibrils, and represents an extremely resistant protection structure for the bacterial colony, possible the strongest naturally synthesized biological material [28,36,37].

Cellulose is a well-studied biomaterial since its discovery in 1838 by Anselme Payen, who determined its elemental composition in oak, beech, aspen, and ambatch, and described it as a fibrous component insoluble in solvents and even alkaline solutions. Cellulose consists in long, linear homopolymeric chains of hundreds to 20,000 d-glucose units, in which the repeating segment, called cellobiose, is a dimer of two β-1,4-linked anhydro-d-glucose units, with every unit corkscrewed 180° towards its neighbors [2]. Neighbor glucose units are covalently linked through acetal bonds between the C1 and C4 carbons of the glucopyranosic rings, and by two intramolecular hydrogen bonds that stabilize the ribbon-shape chain between the C3 and C6 hydroxyls of one glucose unit with the ring oxygen and C2 hydroxyl of neighbor unit [38]. During the biosynthesis of cellulose, each polymer chain has a reducing end which is stabilized as a cyclic hemiacetal, and which points away from the bacterium, and a nonreducing end that was proved to be the place where the chain-growing by cellulose synthase takes place [39]. These macromolecular chains further self-associate—in groups of 36 chains after one proposed model [40], or groups of 24 chains in a second proposed model [41]—by intramolecular hydrogen bonds and intersheets van der Waals interactions between the glucopyranosic rings [38,42] and form a protofibril, while a group of protofibrils becomes a nanofibril. Hundreds of nanofibrils are further assembling in microfibrils that give rise to a resistant tridimensional biofilm floating on soup’s surface. This cellulose-based biofilm represents a highly adaptive microbial macro-colony with a protective role for its community of prokaryotes or eukaryotes, highly resistant even in harsh Mars-like conditions of vacuum, temperature fluctuations, and high UV radiation [36]. 

In the light of these aspects, the preparation of cellulose nano- and microfibrils from KMs could be a valuable technological process that converts a waste into a high-value biomaterial. SCOBY develops well on various polyphenols containing broth, in normal conditions, and without expensive measures intended to maintain culture axenicity. However, brown colored polycondensation products resulted from Maillard reaction, called melanoidins [43], are formed during acetic acid fermentation as compounds embedded into KMs. Therefore, purification of KMs by removing the bacterial and yeast cells and their metabolites, including proteins, peptides and oligosaccharides, is required before further treatments. In the present study, a facile route to obtain the high-valued cellulose nano- and microfibrils by purification of KMs and further mechanical disruption of the cellulosic macro-structure into nanofibrils is shown. We present here the results of three different processes of BNC obtaining from KMs (by microfluidization, atomization, and colloidal milling), the evaluation of the purification degree, the size and aspect of cellulose fibrils after each treatment step, and the physical-chemical properties of intermediary and final products. A comparison of BNC with micro-crystalline cellulose (MC) from wooden sources is also provided in terms of behavior to mechanical treatment, particle size, aspect, and physical-chemical properties.

## 2. Materials and Methods

**Figure 1:**
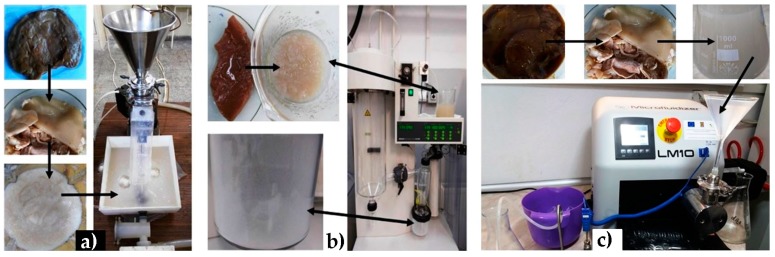
Main steps in purification and nanocellulose (NC): (**a**) purification, grinding with a blender, and deep grinding with a colloidal mill; (**b**) purification and atomization using a spray-dryer; (**c**) purification and microfluidization at pressures over 1300 bar.

The raw cellulosic material was represented by Kombucha membranes, a by-product during the fermentation of tea broth by a symbiotic culture of bacteria and yeast. The tea infusion was produced from 5 g of green tea (Basilur green tea, Ceylon), infused for 15 min into boiling sterile water. The tea infusion was sweetened with 12% high fructose corn syrup (Hungrasweet F50, Hungrana Kft., Ipartelep, Hungary); Hungrasweet have a dry matter content of 75% and contain 50% fructose, 44% glucose and 6% oligosaccharides). Two liters of sweetened infusion were aseptically distributed into a sterile brown glass bottle of 3 liters. The sweetened infusion was inoculated with 10% of previous fermented Kombucha tea, as inoculum. The used symbiotic culture of bacteria and yeast (SCOBY), initially sourced from a Romanian culture, includes acetobacteria, from two genera, *Komagataeibacter* and *Gluconobacter*, yeasts from several genera, mainly *Zygosaccharomyces*, *Brettanomyces*/*Dekkera* and *Pichia*, and lactobacteria. The brown glass bottle was covered with a cotton towel, which was fixed on the neck of the bottle with an elastic band. The Kombucha culture was fermented at room temperature (around 23 °C) for 30 days, for the production of the Kombucha vinegar. The initial, brown, Kombucha membranes represented the sample K_0, and had a weight of 461 g. NaOH in two concentrations (1 and 4 M), using a 1:2 solid:liquid ratio, was tested to purify KM from proteins, saccharides, and amino acids; the samples were encoded K_1M and K_4M. Depending on the thickness of the membrane, the initial membranes were washed with 1 M NaOH solutions 10 to 30 times, 10 min each time, using also an Elmasonic P ultrasonic bath (Elma, Singen, Germany) to intensify the process. After alkaline and ultrasonication treatment, KMs were intensively washed up with distilled water to a neutral pH. The beige-white membranes (K_1M), weighing 347 g, were further treated with the stronger alkaline solution, 4 M NaOH, and were neutralized again with distilled water in excess. Purified K_4M never-dried membranes, weighing 330 g (1% *w* dried cellulose, all concentrations will refer to dry-cellulose content), were subjected to grinding with a blender, 1000 W, 10 series of 5 min, followed by dilution with 5.5 L water (5.7 × 10^−2^% *w*/*v*), the resulting samples being encoded K_B. K_B was further treated by wet deep grinding using a recirculating colloidal mill with 250 µm space between blades, 25 L/min flow rate, for 3 h, which represented about 800 passes (samples encoded K_CM, the process being presented in Figure 1a. Following this step, two different approaches were experimented:

I. Atomization, or spray-drying, of a 10× diluted (5.7 × 10^−3^% *w*/*v*) colloidal mill sample using a Mini Spray dryer B290 (Buchi, Flawil, Switzerland) was used to produce BNC in dried form (samples encoded K_AT, the process being presented in Figure 1b. The dried form of nanocellulose is important for different types of bionanocomposites. The optimized working conditions for nano-atomization were 4 mL/min flow rate for the cellulose suspension, 500 L/h air flow, 175 °C inlet temperature, and 90 °C outlet temperature.

II. Secondly, microfluidization at very high pressure through a ceramic chamber was used to destruct the never-dried microfibrils into nanofibrils and to obtain a water suspension of BNC, respectively MC (process presented in Figure 1c. MC used for comparison with BNC was represented by commercial Avicel. A LM10 microfluidizer (Microfluidics, Westwood, MA, USA) was used, working at 1379 ± 69 bar or 20,000 ± 1000 psi, the implicit units of the equipment. The temperature of cellulose suspension inside the spire, after the exit from the ceramic chamber, was kept under 40 °C using an ice bath. The equipment does not have a recirculation system, so microfluidization was performed for a number of only 1, 10, and 25 passes, the results being still extremely interesting. The resulted BNC samples obtained from KM by microfluidization were encoded K_M1P, K_M10P, and K_M25P, while the MC samples were encoded MC_1P, MC_10P, and MC_25P.

The initial, intermediary, and final samples were analytically characterized by TEM, SEM, DLS, XRD, XRF, FTIR, TGA, and DTG in order to correlate the purity degree, the size and aspect of the particles, and the physical-chemical properties of BNC and MC with the treatment method and working conditions.

For acquisition of images in the scale 20–2000 nm, a transmission electron microscope Tecnai™ G2 F20 TWIN Cryo-TEM (2015-FEI Company™, Hillsboro, OR, USA), working at 30 kV in LFD mode was used. Cellulose samples were easily prepared by pouring a small droplet of aqueous suspension on a holey carbon grid, without staining, due to a sufficient contrast given by the cellulose fibers. Micrographs were obtained using an Environmental Scanning Electron Microscope (ESEM) (FEI-Quanta 200, FEI Company™, Hillsboro, OR, USA)working in low vacuum (1 torr pressure), Large Field Detector (LFD) mode, at 30 KV accelerating voltage, and 12 mm work distance.

Dynamic Light Scattering (DLS) was employed to determine the aggregates’ hydrodynamic average diameter and the size distribution of microfluidized bacterial and microcrystalline cellulose samples in aqueous dispersion using a Zetasizer Nano ZS ZEN 3600 instrument (Malvern Instruments, Malvern, UK) that can determine particle size in the range of 0.6–6000 nm Two concentrations were tested, respectively 5.7 × 10^−4^% *w*/*v*, and 5.7 × 10^−5^% *w*/*v*. The same equipment and same concentrations were used to determine the zeta potential by electrophoretic mobility measurement technique, or Laser Doppler Velocimetry (LDV).

X-ray diffraction data were collected with a Rigaku-SmartLab diffractometer, operating at 45 kV and 200 mA. Measurements were done using Cu_Kα1_ radiation (wavelength 1.54059 Å), in parallel beam configuration, using the following system: the incident parallel slit was 5°, the incident slit was 0.2 mm, length limiting slit was 10 mm, the receiving parallel slit analyzer was 0.5°, and the receiving parallel slit was 5°. The scanning was performed in theta/2theta mode, in 2θ range from 10° to 50°, with a step of 0.02° and scan speed of 4°/min. The degree of crystallinity and the separation of peaks were carried out using the PDXL 2.7.2.0. software (Rigaku Corporation, Tokyo, Japan).

X-ray fluorescence (XRF) measurements were performed using an energy-dispersive spectrometer, EDXRF PW4025, type MiniPal 2 (PANalytical, B.V., Almelo, The Netherlands), with a Si-PIN detector, at 20 kV and automatic current intensity, measurement time 300 s, in Helium atmosphere. Sulfur was quantified using a dedicated calibration curve.

Fourier transform infrared spectra (FTIR) were recorded in the attenuated total reflectance (ATR) technique, ranging from 4000 to 600 cm^−1^, by accumulation of 32 spectra at a resolution of 4 cm^−1^ on a Spectrum GX spectrometer, (Perkin Elmer, Waltham, MA, USA). In order to compare the spectra of bacterial and vegetal cellulose samples, purified and mechanically treated, all FTIR spectra were normalized and corrected for the baseline.

Thermogravimetrical analyses were performed using a TGA Q5000IR (TA Instruments, New Castle, DE, USA) equipment, 100 µL platinum pan, 1–3 mg sample size. The standard TGA mode used nitrogen (99.99%) as balance purge gas, 10 mL/min flow rate, nitrogen (99.99%) 40 mL/min as sample purge gas. Standard TGA Method 1: N_2_, Ramp 10 °C/min to 700 °C, 2: Select gas 2. 3: Isothermal for 5 min. Hi-Res TGA method used nitrogen (99.99%) 10 mL/min as balance purge gas and synthetic air (99.99%) 50 mL/min as sample purge gas. Hi-Res TGA Method: Air, 1: Hi-Res sensitivity 1, 2: Ramp 20 °C/min res 4 to 700 °C. 

## 3. Results

The performed experiments aimed to study four issues regarding the production and physical-chemical properties of bacterial micro- and nanocellulose obtained from KM: 1. Purification of Kombucha membranes; 2. Size and aspect of bacterial cellulose fibrils; 3. Physical-chemical properties of bacterial nano/microcellulose from KM; 4. A comparison with a well-studied micro-crystalline cellulose from wooden sources, Avicel.

### 3.1. Purification of Kombucha Membranes

Three main treatment approaches can be considered for the treatment of cellulose materials: alkaline, acidic, and with particular solvents, like ionic liquids, deep eutectic solvents, supercritical fluids, and others. In our work, we have chosen alkaline treatment for removing melanoidins from Kombucha membranes. “Melanoidins” is a general terminology for the products of non-enzymatic reaction between amino acids and reducing sugars, called Maillard reaction. Melanoidins represent a heterogeneous mixture of negatively charged, nitrogen-containing, high and low molecular weight compounds [43], which have various biological properties like antioxidant, antimicrobial, antihypertensive, and detoxifying activity, and even tumor growth-inhibiting properties [44]. They are also responsible for the particular taste and brownish color of various bakery products, cooked food, coffee, barley malts, and, in our particular case, for the brown color of Kombucha membranes before purification (Figure 1).

Two alkaline solutions of 1 and 4 M NaOH were used in our work to solubilize melanoidins, and ultrasonication was added to intensify the extraction process. It was observed that during the purification process, the thinner membranes suffer a slight degradation, and it is also possible to lose some nanocellulose due to ultrasonication. It was not the aim of the present study to optimize the purification process in terms of extraction solvent, concentration, temperature, frequency and time of ultrasonication, the main aim was to obtain and characterize bacterial cellulose fibrils from KM by-product. Neither the soup, nor the pellicle content were evaluated concerning the type and amount of melanoidins, but a future study is planned to be focused on these aspects.

Initial Kombucha brown membranes weighed 471 g, KM washed with 1 M NaOH weighed 347 g, while KM washed with 4 M NaOH weighed 330 g. During our experiments, we visually observed that a low NaOH concentration did not provide a proper purification of the initial Kombucha membrane, KM having a light-beige color. Further treatment with the 4 M NaOH solution increased the whiteness level of KM and we assumed a purification degree of around 97%.

#### 3.1.1. SEM Analysis

**Figure 2:**
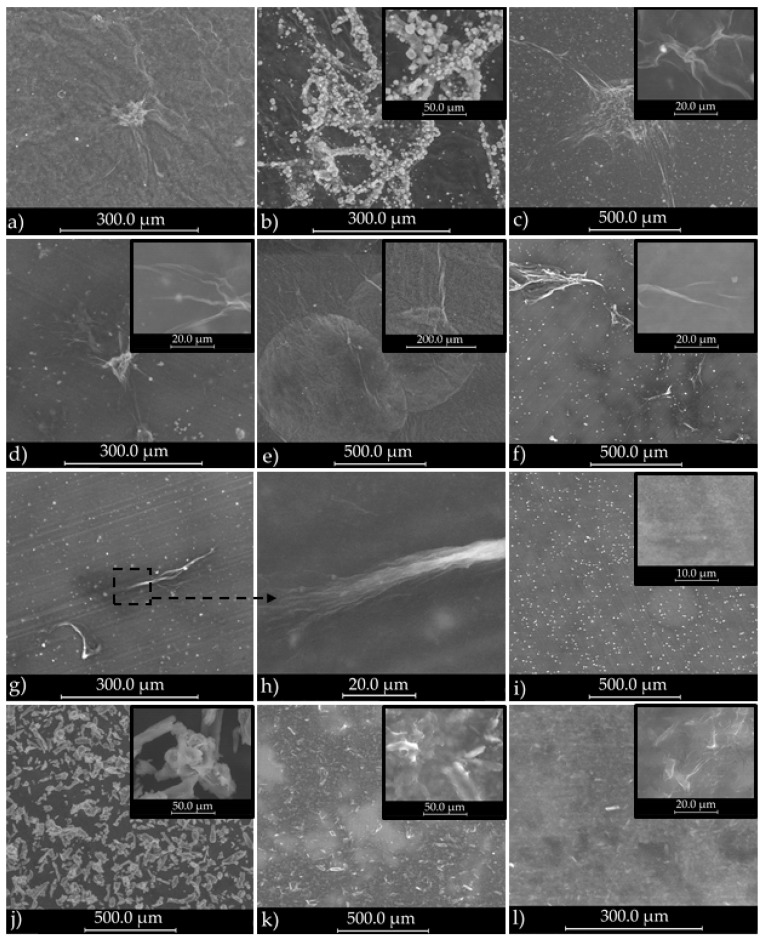
SEM images of biocellulose from Kombucha: (**a**) K_0 = initial membrane; (**b**) K_1M = K_0 washed with 1M NaOH; (**c**) K_B = K_4M after grinding with a blender; (**d**) K_CM = K_B after grinding with a colloidal mill; (**e**) K_AT = K_CM after atomization; (**f**) K_M1P = K_CM after 1 pass through microfluidizer; (**g**) K_M10P = K_CM after 10 passes through microfluidizer; (**h**) K_M10P—disruption of a microfibril into nanofibrils after 10 passes; (**i**) K_M25P = K_CM after 25 passes through microfluidizer; (**j**) MC_0 = vegetal micro-crystalline cellulose; (**k**) MC_1P = MC_0 after 1 pass; (**l**) MC_25P = MC_0 after 25 passes through microfluidizer.

Scanning electron microscopy (SEM) offered important visual information regarding the morphology of cellulose networks, and the modification of matrix structure after each treatment step (Figure 2).

The initial Kombucha membrane (K_0) appears to consist of cellulosic clusters of hundreds of microns surrounded by a homogeneous phase which represent the melanoidins that cover and intercalate through the cellulosic network (Figure 2a). For the Kombucha membrane washed with 1 M NaOH (sample K_1M, Figure 2b), the purification is incomplete due to the low concentration of alkaline solution, since some microcrystals can be seen attached to cellulosic microfibrils. To speculate about the type of these microcrystals, we will refer later in this section to XRD and XRF analyses. From the SEM micrograph presented in Figure 2b) it appears that these microcrystals have an inorganic nature. Further washing with a 4× concentrated NaOH solution leads to what we assumed to be a 97% purification degree. The subsequent light mechanical treatments with a blender (K_B, Figure 2c) and with the colloidal mill (K_CM, Figure 2d) are destructuring the pellicle into cellulosic micro-structures of 0.5–2 mm, respectively 50–500 µm and smaller. Sample K_AT resulted after atomization looks fairly homogeneous in SEM images (Figure 2e), with only few microstructures of 20–50 µm. Deep mechanical treatment at pressures over 1300 bar using a microfluidizer has the effect of further reducing the size of cellulosic structures. If in sample K_M1P, after only 1 pass through the microfluidizer’s ceramic chamber, there can still be seen some cellulose clusters of 100–500 µm (Figure 2f), after 10 passes these clusters are much reduced in number and size (Figure 2g). For the sample K_M10P, a very interesting micrograph (Figure 2h) is showing an intermediary step of the mechanical treatment: a microfibril of 60–100 µm in length, 5 µm in diameter, is defibrating at one end in 8–12 nano-fibrils after 10 passes through microfluidizer. After 25 passes (Figure 2i), no micro-structures of cellulosic fibrils with size larger than 10 µm can be seen, but only 1–2 µm crystals dispersed in a homogeneous phase of what we consider to be predominant cellulosic nanofibrils.

Microcrystalline cellulose was used for comparison with bacterial nanocellulose (Figure 2). MC was submitted to the same number of passes through the microfluidizer and all analytical investigations were performed for all MC samples also. Compared to BNC, SEM images evidenced that MC is made of crystalline clusters of 20–80 µm and also particles with sizes under 20 µm (Figure 2j). After microfluidization, the clusters decrease in dimension to aggregates smaller than 50 µm and particles with sizes under 20 µm (Figure 2k,l).

#### 3.1.2. XRD Analysis

X-ray diffraction (XRD) analyses gave information regarding the cellulose allomorphs, the purification steps, and the influence of mechanical treatments. The values of crystallinity degree (*X*c), Braggs’ angles (2θ), interplanar distances (*d*), and crystallites size (*D*) were determined with PDXL 2.7.2.0 software. Native cellulose, bacterial or vegetal, is type I cellulose composed of two crystal allomorphs, cellulose Iα and Iβ, which are structurally similar. Both cellulose allomorphs have their main structure composed of glucose units, but in bacterial cellulose and some green algae prevails cellulose Iα, which is made of one-chain triclinic unit cells (*t*). In contrast, the complex structure of plants, with cellulose intercalated with hemicellulose, pectin, and lignin, is preponderantly composed of cellulose Iβ that consists in two-chains monoclinic unit cells. Extremely interesting is the fact that cellulose I has never been crystallized in vitro [45], its process of crystallization by arranging 36 [40] or 24 [41] polymeric chains in the same direction being one of the nature’s wonders. Through the intra- and intermolecular hydrogen bonds between glucose units and neighbor chains, and the intersheets van der Waals interactions between the glucopyranosic rings, cellulose Iα and Iβ build together the strongest natural material.

**Figure 3:**
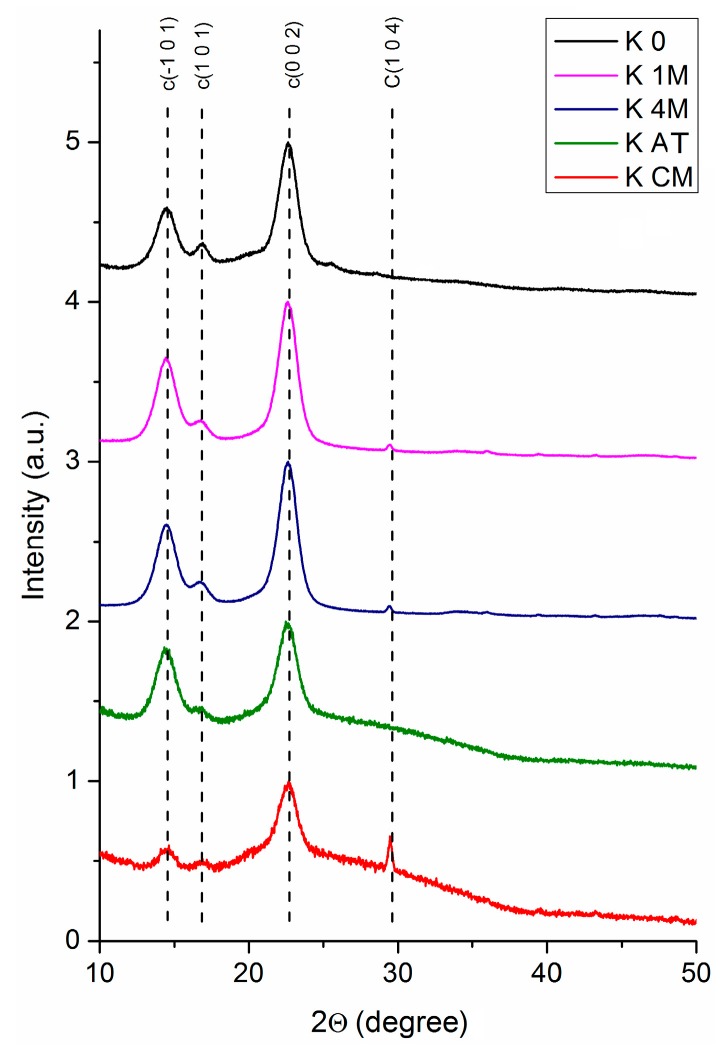
X-ray diffraction (XRD) patterns for Kombucha membranes at different processing stages: K_0 = Kombucha initial membrane; K_1M = Kombucha membrane washed with 1 M NaOH solution; K_4M = Kombucha membrane washed with 4 M NaOH solution; K_B = K_4M after grinding with a blender; K_CM = K_B after grinding with a colloidal mill with recirculation; K_AT = K_CM after atomization; c = cellulose peaks; C = CaCO_3_ impurity. (a.u.—arbitrary units).

**Figure 4:**
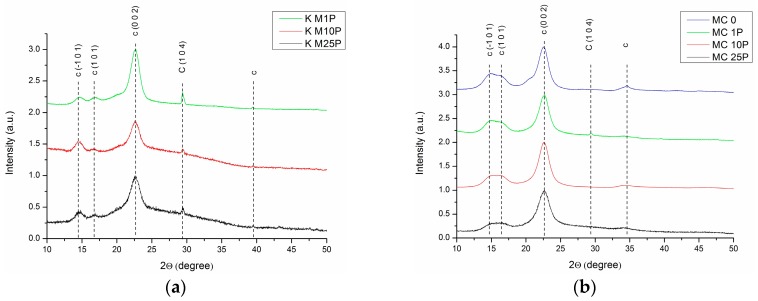
(**a**) XRD patterns of bacterial cellulose from KM at different number of passes through microfluidizer (K_M1P—1 pass; K_M10P—10 passes; K_M25P—25 passes); c—cellulose peaks; C—CaCO_3_ impurity. (**b**) XRD patterns of micro-crystalline cellulose at different number of passes through microfluidizer (MC_1P—1 pass; MC_10P—10 passes; MC_25P—25 passes); c—cellulose peaks; C—CaCO_3_ impurity.

Cellulose is a semicrystalline material, having both crystalline and amorphous regions arranged in a fringed-fibrillar model (Battista, 1950), although some other fringed-fibrillar models, with various shapes were afterwards proposed. XRD analysis is an extremely useful tool to describe this type of material, and captures very interesting aspects of the purification and destructuring of Kombucha membranes into micro- and nanofibrils. Depending on the origin and purity of cellulose, 2 or more peaks can be recorded using XRD [46,47]. The initial Kombucha membrane (K_0) presents 2 main peaks on the diffractogram, at 2θ values of 14.49° and 22.64° (Figure 3). For the cellulose sample with the highest crystallinity, sample K_4M (washed with 4 M NaOH), 5 main peaks are visible on the diffractogram, while for intermediary samples like the sample grinded with the colloidal mill (K_CM), the number of peaks decrease to 4, and further to 3 for the most intensively microfluidized sample, K_M25P (Figure 4). The decrease of XRD peak intensity and peak broadening are generally explained by two overlapping phenomena: increasing of amorphous phase as a result of various treatment methods, and, secondly, decreasing of crystallites’ size and of their ordered arrangement [47]. For the analyzed samples, the main peaks appear at 2θ values of 14.48° ± 0.43°, 16.60° ± 0.26°, 22.61° ± 0.06°, and 34.6° (Figure 3 and Figure 4 ). The main values (e.g., 14.48°) represent the mean of extremes (minimum and maximum), while the secondary values (e.g., ± 0.43°) express the peaks’ shifting range following the various treatment methods applied in this work. 

Phase identification by XRD was accomplished using the characteristic diffraction peaks found in Power Diffraction Files (PDF) from the International Centre for Diffraction Data (ICDD, Newtown Square, PA, USA). For cellulose Iβ the 2θ values of 14.83°, 16.42°, 22.71° (Table 1) were attributed to (−101), (101), and (002) diffraction planes, and the broad peak around 34.6° to a convolution of 5 diffraction planes (ICDD: 00-060-1502). Also, 2θ values of 14.26°, 16.77°, 21.80°, and 39° where assigned to diffraction planes (−101), (010), (−110) and (103), characteristic for cellulose Iα (ICDD: 00-056-1719). The most intense maximum diffraction (104) of calcite can be observed at 2θ value of 29.52° (ICDD: 01-072-1650). In literature, there were identified diffraction peaks characteristic for cellulose at 2θ values of 14.7°, 17.0°, 22.9°, 34.6°, attributed to the diffraction planes (101), (101), (002), and (040) [48], or at 15.1° (1–10), 16.5° (110), 22.5° (200), and 34° (004) [49].

Shifts in Bragg’s angle and variation of interplanar distances can offer additional information regarding the changes in the morphology of cellulosic materials as a result of different chemical and mechanical treatments. Shifts of 2θ angles to higher values, so to lower d-spacing values, signify an increase in strength [50], which means that shifting to higher angles, in our case, signify a stiffening in cellulose structure due to mechanical treatment, and also an enrichment in Iβ allomorph towards Iα. Also, the *Z* criterion calculated in Table 1 according to [50] offers information on the rapport between cellulose I allomorphs, indicating a predominance of cellulose Iα if *Z* > 0, or of cellulose Iβ if *Z* < 0. Indeed, correlating this criterion with the fact previously underlined, that cellulose Iα is predominant in bacterial cellulose (K-samples) and algal cellulose, while Iβ in plant cellulose (MC-samples), the data in Table 1 confirm this aspect, with one exception for sample K_M1P. The value of *Z* criterion for K_M1P sample, −2.223, is small and it may not be relevant to draw a conclusion that sample K_M1P was enriched in Iβ after 1 pass through the microfluidizer. 

Crystallinity degree (*X*c) can be calculated by various methods, and debates are still undergoing regarding the most rigorous method [46,47]. In our study it was employed the method of intensities’ ratio, respectively *X*c = *I*_crystlline_/(*I*_crystalline_ + *I*_amorphous_), and the results are presented in Table 1 for both, BNC and MC samples. The initial, brown, Kombucha membrane (K_0) has a low crystallinity, 37%, due to the presence of melanoidins all around and inside the cellulose matrix, besides the amorphous cellulose. The first purification step with a low NaOH concentration, 1M (sample K_1M), removes a high amount of proteins, bacteria, amino acids, and other fermentation residues, which leads to an increase in crystallinity to 80%. As the visual aspect of the cellulose suspension suggests, being slightly beige instead of white, the removal of melanoidins after this step is incomplete, fact confirmed by SEM micrographs, and by other analytical methods further presented. Washing with a more concentrated NaOH solution, 4 M (sample K_4M), leads to a deeper purification of cellulosic membranes. XRD analysis confirms this aspect by an increase of crystallinity to 87%, the sample having also a mat-silver-white color. Sample K_4M was further processed by mechanical grinding with a blender (K_B) and with a colloidal mill (K_CM). Mechanical treatments led to a drastically decrease in crystallinity, from 87% to 42.5% for sample K_B, and to 21.5% for sample K_CM. This drastically decrease in crystallinity suggests that the initial, ordered, highly crystalline cellulosic structure build by bacteria is being defragmented and disordered by mechanical treatments [49]. K_CM sample was further treated by two different approaches: (i) atomization (or spray-drying), that leads to a powder form of bacterial cellulose, and (ii) deep, wet mechanical treatments by microfluidization at pressures over 1300 bar of the so-called never-dried bacterial cellulose. Subjecting the samples to atomization process, which implies spraying to a micro-nozzle, led to an almost simultaneously rearrangement and drying of micro- and nanofibrils, evidenced by an increase in crystallinity to 63%. The other approach, microfluidization at high pressure, implies the flow of the diluted cellulose suspension at pressures over 1300 bar through a tortuous tunnel in a ceramic chamber. The suspension exits at high pressure through a narrow nozzle which continues with a 30 cm length steel spire of 200 µm internal diameter. The fact that crystallinity increases with the number of passes through the microfluidizer, from 21.5% for the sample before microfluidization, to 28% after 1 pass, 42% after 10 passes, and 46% after 25 passes, suggests that the microfluidizer system has two roles: 1. to destruct the cellulosic macro- and microstructures, at high pressure, inside the tortuous ceramic chamber; 2. to partially rearrange the resulting micro- and nanofibrils by exiting at high pressure through the narrow nozzle and flowing through a 200 µm diameter spiral tunnel.

#### 3.1.3. XRF Analysis

To the best of our knowledge, X-ray fluorescence analysis is applied for the first time to evaluate the elemental composion of bacterial cellulose samples (especially their content in melanoidins). Regarding the use of XRF analysis for cellulose samples, there are only a few studies on this subject, mainly related to metal catalysis of cellulose-based materials [51,52]. XRF method has some limiations because only ellements with the atomic number between 11 and 92 can be identified.

**Figure 5:**
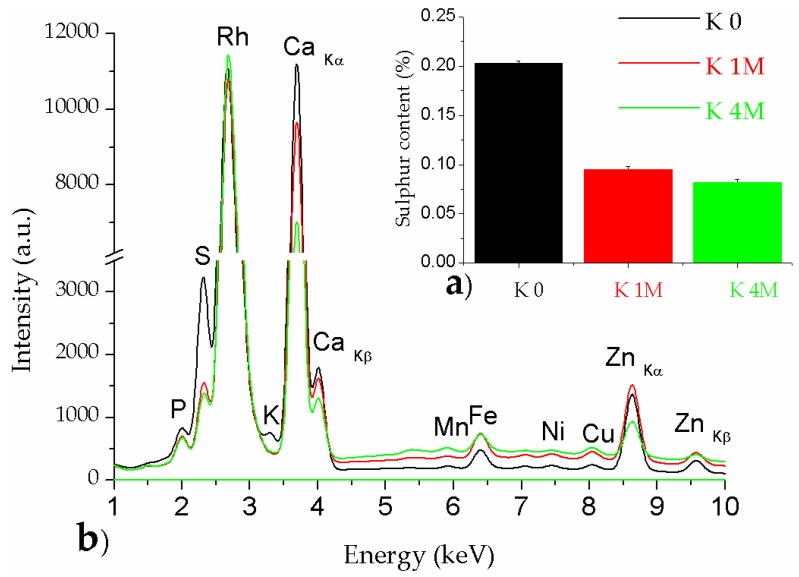
X-ray fluorescence (XRF) analyses: (**a**) sulfur content; (**b**) mineral content.

In our case, XRF analysis offered important information regarding the elemental content in the initial Kombucha membrane, and the evolution of elements’ abundance after the two purification steps of cellulose materials. Initial membrane (K_0) contains relatively high amounts of S, Ca, and Zn, while in lower amounts the elements P, K, Fe, Mn, Ni, and Cu are present (Figure 5). If we correlate the capacity of melanoidins to chelate metal ions [43,53], we can see another confirmation of melanoidins removal in the decreasing of all mentioned elements after washing with alkaline solution. Moreover, it was shown previously that XRD analysis of sample K_1M evidenced a diffraction peak at 2θ = 29.36° characteristic for the diffraction plane (104) of calcite (CaCO_3_). XRF analyses confirmed that after washing KM with the low NaOH concentration, Ca still remains in a high proportion inside membrane K_1M. These findings suggest that the 1 M NaOH solution washed preponderantly the proteins, amino acids, and other types of organic matter susceptible to low chemical stability, while a significant amount of minerals remains attached to the cellulose microfibrils. The previous assumption that the 4 M NaOH solution achieved a 97% purity was correlated here with the sulphur content. Quantitative analysis was performed using a dedicated calibration curve for sulphur, and based on the fact that S can be found in the aromatic thiols bound by melanoidins [54,55]. Based on these assumptions, the purification degree after washing with 1 M NaOH was evaluated by weight loss to 85%, and by sulphur loss to 87%.

### 3.2. Size and Aspect of Bacterial Cellulose Fibrils

#### 3.2.1. TEM Analysis

TEM images evidenced the size and aspect of bacterial cellulose after purification and mechanical destructuration of Kombucha membranes. A first remark is that the treatment with 1 M NaOH solution successfully dissolved a large amount of melanoidins, but did not completely removed them. Remaining melanoidins appear in TEM micrographs as a uniform, light gray pellicle covering the cellulosic, fibrous matrix, marked with white arrows in Figure 2a,b. Secondly, the 4 M NaOH treatment, followed by grinding with a blender, with a colloidal mill, and spray drying led to a partial nanoatomization, cellulose nanofibrillar structures being visible in the sample analyzed by TEM (Figure 2c). Microfluidization at pressure over 1300 bar led to a gradually increase in nano-fibrils content from 1 pass to 25 passes (Figure 2e,g,i), but more passes seem necessary for a higher amount of nanofibrils. For comparative purposes, micro-crystalline cellulose (MC) was treated by microfluidization in the same conditions. MC also suffered a size reduction in crystallites’ size, as can be seen by comparing Figure 2f,h. Interestingly, round-shaped clusters of CNC were obtained after.

**Figure 6:**
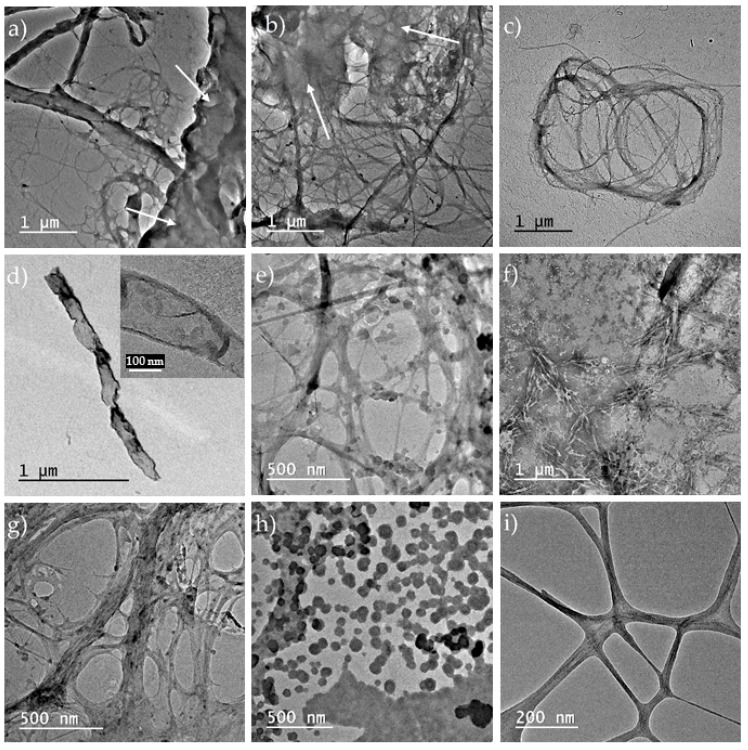
Transmission electron microscopy (TEM) images of cellulose fibrils: (**a**) initial Kombucha membrane (K_0); (**b**) after 1 M NaOH washing (K_1M); (**c**,**d**) after 4 M NaOH washing and nano-atomization (K_AT); (**e**) after 1 pass through microfluidizer (K_M1P); (**f**) micro-crystalline cellulose after 1 pass (MC_1P); (**g**) K_CM after 10 passes (K_M10P); (**h**) micro-crystalline cellulose after 10 passes (MC_10P); (**i**) K_CM after 25 passes through microfluidizer (K_M25P).

For our samples of bacterial cellulose from KM, respectively MC, TEM images presented in Figure 6 evidenced a gradually destruction of macro and micro-structures of cellulose by intensity-increasing mechanical treatments, from grinding with a blender, followed by grinding with a colloidal mill and ending with 1, 10, and 25 passes through a microfluidizer at pressures over 1300 bar. As can be seen from in Figure 6, this cascade of mechanical treatments is destructuring the initial pellicle (K_0) into cellulosic micro-structures of 0.5–2 mm with the blender (K_B), to 50–500 µm and nanofibrils with a colloidal mill (K_CM), to smaller than 100 µm after 10 passes (K_M10P), and smaller than 10 µm after 25 passes and a high proportion of nanofibrils (K_M25P). A similar decrease in particles’ size is observed for MC samples also (Figure 6f,h).

#### 3.2.2. DLS Analysis

Dynamic Light Scattering might be considered an inappropriate analytical method to evaluate the dimensions of cellulose nano-/microfibrils, since they are not particles with a relative sphericity. This aspect can be seen in Table 2, where the DLS results of bacterial cellulose samples and microcrystalline cellulose samples after different number of passes through the microfluidizer were presented. Some of the bacterial cellulose samples have high average diameter and high polydispersity index. In contrast, microcrystalline cellulose, the wooden-based samples (encoded MC_1P, 10P or 25P depending on the number of passes), are 2–20 nm in diameter, 100 nm–1–2 µm in length particles that agglomerate during drying process in micronic clusters of 400–900 nm, as can be seen in Table 2. All samples were analyzed at two concentrations, respectively 5.7 × 10^−4^% *w*/*v* and 5.7 × 10^−5^% *w*/*v*, respectively.

Bacterial fibrils are generally considered as bacterial nanofibrils even if they have the length of a few microns, the nano-dimension being their diameter. DLS analyses evidenced a general trend of transition from monomodal to bimodal pore size distribution, clearer at the lower concentration of sample preparation, which we interpret as a new population of nanofibrils with average diameters of 50–200 nm that appears following the mechanical disruption of microfibrils after multiple passes through microfluidizer. If for the bacterial cellulose there can still be observed populations with average diameters of 1–2 µm, which is the length of nanofibrils, for the microcrystalline cellulose the decreasing trend of average diameters from 600–900 nm to 60–100 nm, and also the monomodal to bimodal transition are better evidenced since MC forms nano- and microclusters with a higher relative sphericity. Moreover, following microfluidization, Zeta potential shows a trend to more negative values, which, correlated with the known electronegativity of cellulose, suggests an increase in negatively charged fragments due to mechanical disruption. For microcrystalline cellulose, the most negative value was determined after 10 passes through the microfluidizer.

### 3.3. Physical-Chemical Properties of Bacterial Nano/Microcellulose from KM

Due to its mechanical strength, nanocellulose is of high interest as nanofiller or as component in nanocomposites, where even small amounts less than 5% drastically increase the resistance of final materials [11,18,56,57]. Besides mechanical strength, other physical properties made cellulose and its nanoderivatives to be the most studied biopolymer: the high aspect ratio, high surface area of nanostructured forms, low thermal expansion, flexibility (especially for bacterial nanofibrils), transparency, polar nature and hydrophilicity, susceptibility to magnetic, electric, and shear field, large piezoelectric response for cellulose nanocrystals, at which can be added biocompatibility, low toxicity, and renewability [1,10,58].

In our work, we evaluated some physical-chemical properties of the obtained bacterial nanocellulose from Kombucha membranes, like aspect ratio, hydrophilicity, crystallinity, polar nature, and thermal stability.

#### 3.3.1. TEM, XRD, and DLS Results on Physical-Chemical Properties

From TEM micrographs results, the aspect ratio of cellulosic nano- and microfibrils was correlated to the preparation methods, by spray-drying and microfluidization. By spray-drying were observed two types of bacterial cellulose shapes: a ribbon shape microfibril with 100 nm diameter and 2 µm length (Figure 3d), and a cellulose ball of nanofibrils with 5–20 nm fibril-diameter and 2 µm ball’s diameter (Figure 3c). By microfluidization of never-dried bacterial cellulose, there were obtained after 25 passes small networks of nanofibrils with 2–10 nm in diameter (Figure 3i). Hydrophilicity was found to be approx. 1 g BNC:100 mL water, determined after samples of stabile aqueous suspension were oven-dried at 70 °C for 3 days.

Likewise, XRD analyses results were correlated to the intensity of the mechanical treatments. The highest overall crystallinity, 87%, corresponded to the highest purity Kombucha membrane, K_4M. We assume that this high overall crystallinity is related in principal to the ordered structure the bacteria are building, which can be seen as a “bacterial crystal palace”. By intensive mechanical treatments, this “bacterial crystal palace” is broken down into “crystal pieces” that, although they have individual high crystallinity, due to a higher disorder they scatter the X-ray and they record a lower overall crystallinity (21% was the lowest, after grinding with a high-power blender). Through microfluidization, the crystallinity showed a slight increase with the number of passes, from 28% after 1 pass, to 42% after 10 passes, and 46% after 25 passes, fact that we interpreted as a slight rearrangement of nanofibrils after the exit at 1300 bar from the ceramic chamber through a micro-nozzle and micro-diameter tunnel.

Bacterial fibrils are generally considered as bacterial nanofibrils even if they have the length of a few microns, the nano-dimension being their diameter, of 20–100 nm. DLS analyses evidenced a general trend of transition from monomodal to bimodal pore size distribution, clearer at the lower concentration of sample preparation. This may be interpreted as a new population of nanofibrils with average diameters of 50–200 nm that appears following the mechanical disruption of microfibrils after multiple passes through microfluidizer. If for the bacterial cellulose there can still be observed populations with average diameters of 1–2 µm, which is the length of nanofibrils, for the microcrystalline cellulose the decreasing trend of average diameters from 600–900 to 60–100 nm, and also the monomodal to bimodal transition are better evidenced. This is because MC forms nano- and microclusters with a higher relative sphericity.

The negative polarity of BNC was evaluated using the Zeta potential. Following microfluidization, Zeta potential shows a trend to more negative values, which, correlated with the known electronegativity of cellulose, suggests an increase in negatively charged fragments due to mechanical disruption. For microcrystalline cellulose, the most negative value was determined after 10 passes through the microfluidizer. It was determined a trend towards a more negative potential with the number of passes, from −10.4 mV after 1 pass, to −13.1 mV, which can be explain by the breaking of multiple hydrogen bonds and appearance of new nano- and micro-cellulosic fragments with additional free negative potential.

#### 3.3.2. FTIR Analysis

**Figure 7:**
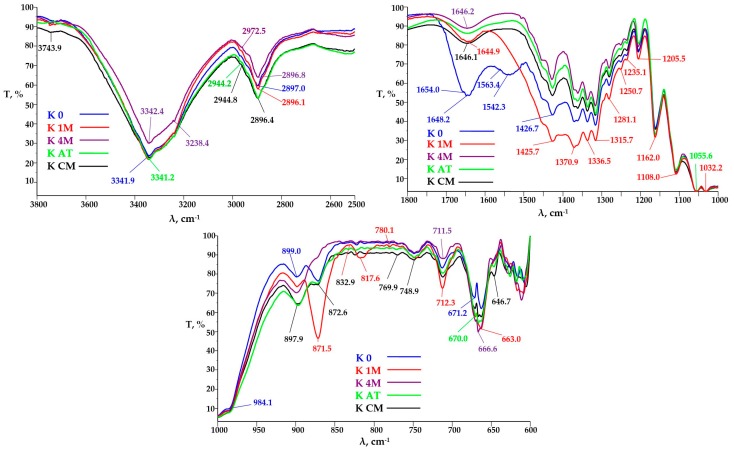
Fourier-transform infrared spectroscopy (FTIR) spectra of KM before and after purification and light mechanical treatments: K_0 = initial KM; K_4M = KM washed with 4 M NaOH; K_AT = K_4M after atomization.

FTIR spectra acquired in the range 4000–600 cm^−1^ offered information about the molecular structure of Kombucha-derived cellulosic materials and corresponding physical-chemical properties. Three areas of interest were analyzed in the FTIR spectra of these materials: from 3880 to 2380 cm^−1^, from 1800 to 1000 cm^−1^, and from 1020 to 600 cm^−1^. The transmittance from 3000 to 3600 cm^−1^ is characteristic to the hydroxyl groups involved in the inter- and intra-molecular hydrogen bonds [59,60]. The band for at 3726–3743 cm^−1^, characteristic to the vibration of the “free” OH group of the bound water [61], has the highest value for K_CM, the increased amount of bound water may be due to the higher proportion of nanocellulose resulted from the mechanical grinding with the colloidal mill. The transmittance bands at 2980 cm^−1^, from 2973 cm^−1^, from 2962 cm^−1^, from 2945 cm^−1^ and from 2870 cm^−1^ characteristic to C–H stretching of –CH_3_ group and 2934 cm^−1^ and 2844 cm^−1^ C–H bond in –CH_2_ are strong in K_AT, which suggest the occurrence of new chain-ends due to the shortening of cellulosic fibrils also by atomization (Figure 7). For sample K_0 there can be observed two transmittance bands at 1654 and 1648 cm^−1^, both specific to C=O in amide I, and a band at 1542 cm^−1^ specific to NH groups in amide II, all three bands confirming the presence of proteins in Kombucha initial membrane [62]. Starting with sample K_1M these bands disappear, which suggest the removal of a high content of proteins in the purification step 1, purity degree determined in Section 3.1. to be 85–87%.

**Figure 8:**
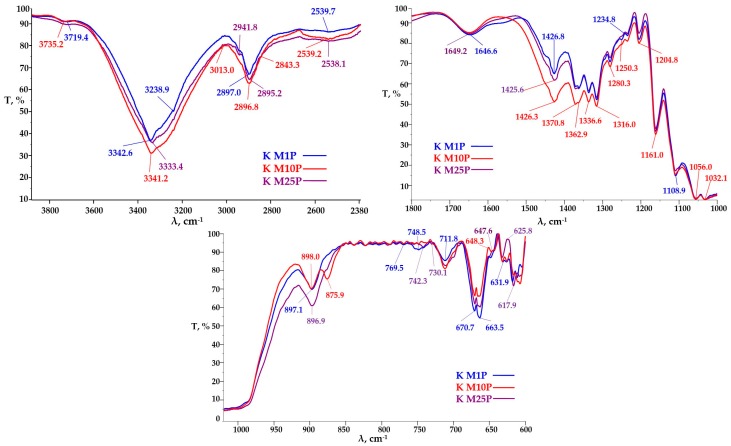
FTIR spectra of KM after different number of passes through microfluidizer.

For the microfluidized samples of KM (Figure 8), the broad transmittance band in the wavelengths interval 3500–3200 ± 50 cm^−1^ characteristic for –OH stretching vibration in strong intra- and intermolecular bonds is wider, ranging from 3650 to 3000 cm^−1^. This is due to the fact that the number of available –OH groups in bacterial cellulose is greater for bacterial cellulose compared to plant cellulose [63]. The transmittance band for –OH at 3735 cm^−1^, characteristic for the “free” OH group of the bound water, has the same transmittance value for K_M1P and K_M10P samples, but a higher value for K_M25P, which means that after a larger number of passes through microfluidizer, the proportion of nanocellulose increased, leading to a higher amount of absorbed water (Figure 8). The absorption bands at 2941 and 2843 cm^−1^ characteristic for the C–H bond in –CH_3_ and –CH_2_ groups occur at K_M10P, but especially at K_M25P, which means the occurrence of new chain-ends due to the shortening by microfluidization of the cellulosic fibrils.

A significant increase of the absorption band transmittance at 1426 cm^−1^ assigned to the C–H bending [64] and of the absorption band at 1371 cm^−1^ also specific for C–H deformation [65] and at 1336 cm^−1^ characteristic to OH in-plane deformation [65] was observed for the K_M10P sample compared to K_M1P and K M25P_samples.

This suggest some differences in the structure of cellulose fibrils. The transmittance bands at 1280 and 1235 cm^−1^, characteristic for C–O–C symmetric stretching and OH in plane deformation are decreasing in the order K_M10P > K_M1P > K_M25P, which suggests that this functional bounds are strongly influenced by the number of passes through microfluidizer. Same phenomenon is observed also for the functional groups –C–O, –C–O–C and–O–H present in the cellulose structure and with characteristic bands at the wavelengths 1205, 1161, and 1110 cm^−1^ [66], which shows the influence of the mechanical treatment conditions. The transmittance values of the bands of–C–O and/or –C–C– and/or –C–C–O groups at the wavelength 1056 and 1032 cm^−1^ are actually equal, which means that these types of cellulose bounds and their afferent vibrations are not influenced by deep mechanical treatment at high pressures. The β-glycoside bond from 897 cm^−1^ is significantly influenced by the number of passes through microfluidizer, the most intensive absorption band being for K_M25P sample, while for the K_M1P and K_M10P samples, the absorption intensities are equal (Figure 8). It is noteworthy that after 10 passes, a vibration band at 876 cm^−1^ is assigned to the pyranosic chain breakage, which is no longer found in the sample with the maximum number of passes, K_M25P.

**Figure 9:**
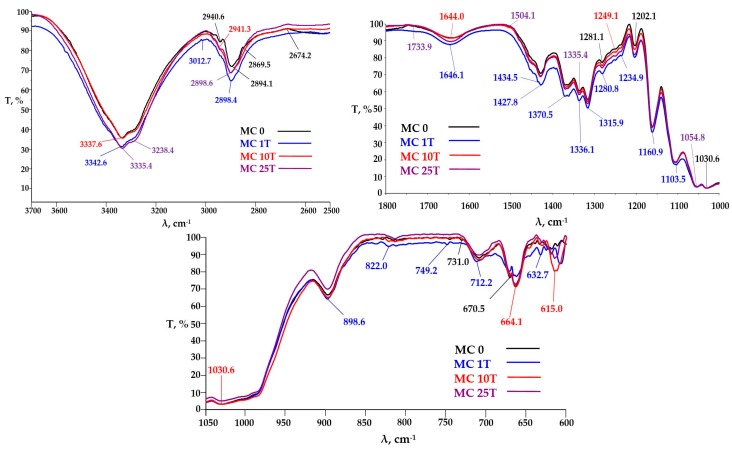
FTIR spectra of micro-crystalline cellulose (MC) samples after different number of passes through microfluidizer.

FTIR analyses for MC samples (Figure 9) evidenced that the transmittance bands of –OH at 3746, 3743, 3734, 3726 and 3714 cm^−1^, characteristic for intermolecular hydrogen bonding, have a high transmittance value for MC_1P compared with MC_10P and MC_25P, which means that a larger number of passes through the microfluidizer leads to the destruction of the intermolecular hydrogen bonds from the microcrystalline cellulose. Transmittance bands at 2968, 2942 and 2870 cm^−1^ characteristic for C–H bond in the –CH_3_ group occur primarily in the MC_0 sample, and less in the MC_25P, whose transmittance is virtually equal with MC_10P. This means that the occurrence of chain lengths due to shortening cellulosic fibrils is not so much influenced by the number of sample passages, but it should be greater or equal to 10. Noteworthy is that the band transmitters at 2899 cm^−^^1^ attributed to –CH_2_ decrease in the order of: MC_1P > MC_10P = MC_25P > MC_0, which confirms the microcrystalline structure breakage and the occurrence of new –CH_3_ groups, therefore the appearance of cellulosic fibril ends is accompanied by a decrease in the transmittance of the –CH_2_ group, thus by the decreasing of cellulosic chain transmittance. The transmittance of band 1646 cm^−1^ decreases in the order MC_1P > MC_25P > MC_10P = MC_0, characteristic for the –OH and/or H–C=C–H bond, i.e., of unsaturation, leads to the conclusion that there is an optimal number of passes through microfluidizer, namely 10, for which the structure of the fibers is most strongly influenced by the applied treatment, fact noticed also by other authors [67].

#### 3.3.3. Thermal Analyses

Thermal analyses offered additional information regarding the purification of initial Kombucha membrane, the destructuration of macro- and micro- cellulosic structures to nanofibrils, and the thermal stability and behavior of Kombucha-derived cellulosic materials after different purification and treatment steps.

**Figure 10:**
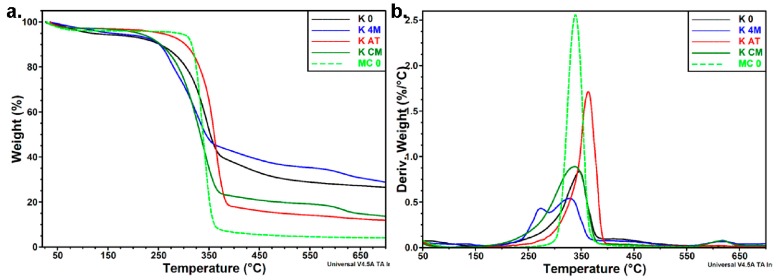
Thermogravimetric analysis (TGA) (**a**) and derivative thermogravimetry (DTG) (**b**) curves in inert atmosphere of Kombucha pellicles.

Thermogravimetry analysis (TGA) and derivative thermogravimetry (DTG) data in inert atmosphere for the initial and different treated Kombucha membranes are shown in Figure 10 and the thermal behavior of MC_0, Avicel untreated microcellulose, is shown for comparison. All the samples showed a first weight loss of 3–6% up to 150 °C because of the volatilization of free and bound water. The weight loss is similar for MC_0, K_AT and K_CM and close to 3%, but higher for K_0 and K_4M, suggesting a higher amount of water retained by these samples, and a higher content of thermally sensitive compounds (especially melanoidins in K_0). The main degradation process, which results from the depolymerization, dehydration, and decomposition of cellulose, occurs within different limits depending on the treatment. The lower thermal stability of most K samples compared to microcrystalline cellulose reference may be due to the thermally labile melanoidins residues and other non-cellulosic products imbedded in the Kombucha pellicles.

The values of the onset degradation temperature (*T*_on_) and the temperature at the maximum degradation rate (*T*_d_) from Table 3 show that both alkaline (K_4M) and mechanical treatments (K_CM) decreased the thermal stability of K_0. This influence is significant in the case of K_4M, a difference of about 30 °C in *T*_on_ and of more than 50 °C in *T*_d_ values being observed. The analysis of DTG curves showed that the main degradation process of K_4M is a two-step process, with a low-temperature shoulder at 273.9 °C and a peak at 328.3 °C, close to the *T*_d_ values of K_CM (338.0 °C) and MC_0 (339.3 °C) but lower than that of the initial K_0 pellicle (346.6 °C). The decrease of the thermal stability of cellulose due to mechanical and chemical treatments was already signaled [49,68]. The higher surface area of cellulose with a nanometric dimension and the larger number of new free ends of cellulose chains were considered as the main causes of this behavior. Moreover, K_4M showed the highest XRD crystallinity (87%) of all the treated or untreated KM samples, and earlier reports have shown that crystallized cellulose chains are efficient pathways for the heat transfer, producing better thermal conductivity and decreasing the thermal stability [69].

Also interestingly, both *T*_d_ (363.5 °C) and *T*_on_ (329.4 °C) of K_AT are higher than the ones of the other treated samples, and even higher than the two references, K_0 and MC_0. The only difference between the sample with the lowest and the highest thermal stability was the atomization step. It can be assumed that the spray-drying process, with an inlet temperature of 175 °C, was able to remove the residual 3% volatile matter that consists in melanoidins-like polycondensation products, which may catalyze the degradation of cellulose. Indeed, the weight loss of K_AT was very small up to 230 °C, under 5%, and similar to that of MC_0, Avicel microcrystalline cellulose, showing that the volatile products were already removed from this sample.

A smaller amount of char residue was noted for all the samples compared to K_0 and K_4M (Table 3). It has been reported that the increased split hydrogen bonds due to the applied mechanical treatments reduce the char residue, which may be an important cause in our case also [70,71]. Another recent report sustains this hypothesis, but it considers also the possibility that the highly polar alkaline ions may catalyze the degradation of glycosidic linkages [49]. Considering the large char residue of K_4M, probably with the highest number of adsorbed alkaline ions, we consider that this last statement is not valid here. It should also be noted that the combined alkaline and mechanical treatments have successfully removed many of the non-cellulosic components and melanoidins, as revealed by SEM, TEM, XRD, FTIR and XRF results, which may also explain a diminished char residue for purified samples.

**Figure 11:**
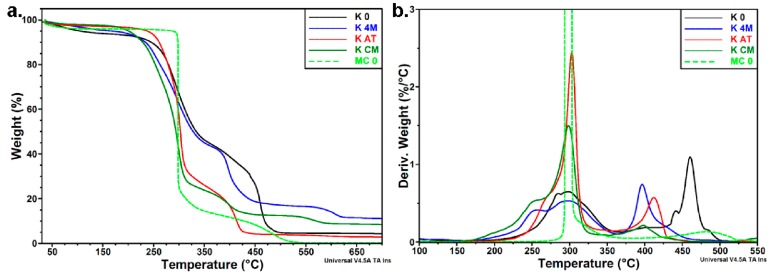
High-Res TGA (**a**) and DTG (**b**) curves of Kombucha pellicles.

The data obtained by high resolution TGA in air (Figure 11) may provide additional information on the degradation process of Kombucha-derived cellulosic materials. 

The High-Res TGA curves (Figure 11a) showed important differences between the degradation processes in air in the case of Kombucha-derived cellulosic materials compared to microcrystalline cellulose. The thermo-oxidative degradation of cellulose takes place as a two-step process, with the main degradation peak at 298 °C and a second maximum at 481 °C, corresponding to the oxidation of the char residue to lower molecular weight gaseous products. The thermo-oxidation of Kombucha-derived cellulosic materials starts at a lower temperature compared to MC_0, regardless the treatment. Several maxima and shoulders were observed in the DTG diagram of K_0. The degradation process that starts from about 200 °C, with a double peak at 284 °C/295 °C, corresponds to the thermo-oxidation or decomposition of thermally labile non-cellulosic matter in the initial K_0 pellicle, overlapped with the depolymerization, dehydration, and decomposition of glycosyl units. A very broad peak around 400 °C and a double peak with a shoulder at higher temperature may be related to the thermal degradation of lipopolysaccharides from bacterial cells and to proteins like chitin, besides the oxidation of the char residue. Three maxima were apparent in the DTG diagram of K_4M (Figure 11b). The two close peaks at 258 °C and 295 °C corresponding to the degradation of non-cellulosic matter and cellulose, respectively, are more apart than in the initial pellicle due to the partial extraction of non-cellulosic products in K_4M. The thermal degradation of more thermally stable non-cellulosic components and the oxidation of the char take place at a lower temperature, probably because of the catalytic effect of highly polar alkaline ions. Similar behavior was noted for K_CM, with the difference that the peak corresponding to the degradation of cellulose was more intense, while that assigned to the degradation of thermally stable non-cellulosic components was very weak, due to the higher purity of this sample. The DTG curve of K_AT denotes a thermal behavior closer to that of MC_0, with a degradation peak at a higher temperature than MC_0 (303 °C compared to 295 °C) and a very small shoulder at a lower temperature, showing small amount of un-extracted non-cellulosic products (which we hypothesized to be around 3%). 

**Figure 12:**
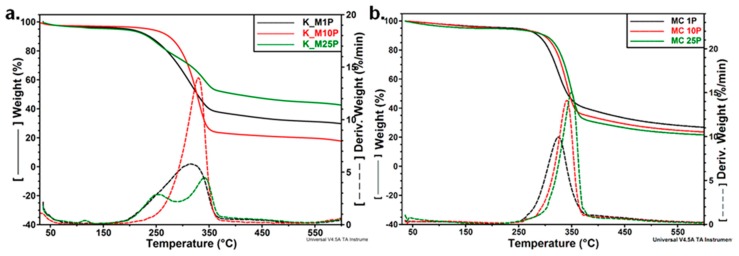
Influence of the passes through microfluidizer on the thermal stability of Kombucha pellicles (**a**) and microcrystalline cellulose (**b**).

The influence of the passes through microfluidizer on the thermal stability of Kombucha-derived cellulosic materials is shown in Figure 12a. Unlike microcrystalline cellulose, where a higher number of passes through microfluidizer led to a gradually increase of thermal stability (Figure 12b), Kombucha materials are less influenced, probably because of the presence of non-cellulosic products.

## 4. Conclusions

Bacterial cellulose membranes, a side-stream product of Kombucha beverages obtaining by fermentation with a symbiotic culture of bacteria and yeast, was found to be a valuable raw material for bacterial nanocellulose (BNC) manufacturing. After alkaline purification to remove the fermentation residues, intensity-increasing mechanical treatments were applied for destructuring the cellulose matrix into high-valued cellulose nano- and microfibrils. The end processes were atomization to obtain dry BNC, and microfluidization to obtain never-dried BNC. TEM, SEM, DLS, XRD, XRF, FTIR, TGA, and DTG were used to gain insight on the initial, intermediary processed, and final cellulose structures. Gradually decrease in size from macro- to micro- and nanodimensions were confirmed by TEM and SEM micrographs, by DLS and Zeta potential, and by an apparent decrease in overall crystallinity of disarrayed nano-sized samples analyzed by XRD. FTIR analyses evidenced new intermolecular hydrogen bonds and new chain-ends formation as a result of profound cellulose matrix destructuration, and also suggested the existence of an optimal number of mechanical treatments. TGA results confirmed the efficiency of the purification process and highlighted a higher thermal stability for spray-dried BNC compared to pure cellulose. Future studies will be focused on optimizing the purification process to a greener and faster method, to a deeper understanding of the bacterial cellulose biosynthesis, and to nanocellulose functionalization to obtain devices for different applications. 

## Figures and Tables

**Table 1:** XRD parameters: crystallinity degree (*X*c), 2θ values, interplanar distances (*d*), and *Z* criterion.

Sample	*X*c	2θ_1_ (°)	*d*_1_ (Å)	2θ_2_ (°)	*d*_2_ (Å)	2θ_3_ (°)	*d*_3_ (Å)	*D* (nm)	*Z*
K 0	37%	14.49	6.104	16.84	5.260	22.64	3.922	6.060	+9.955
K 1M	80%	14.47	6.115	16.74	5.289	22.58	3.933	5.672	+9.202
K 4M	87%	14.53	6.081	16.80	5.273	22.59	3.931	5.713	+4.889
K AT	63%	14.50	6.101	16.83	5.261	22.61	3.928	5.520	+9.357
K B	42.5%	14.05	6.296	16.86	5.253	22.61	3.929	5.981	+43.092
K CM	21.5%	14.55	6.082	16.85	5.256	22.66	3.920	5.664	+6.591
K M1P	28%	14.67	6.031	16.84	5.258	22.61	3.928	6.228	−2.223
K M10P	42%	14.56	6.076	16.67	5.311	22.59	3.932	6.030	+0.615
K M25P	46%	14.51	6.096	16.70	5.302	22.60	3.930	5.312	+4.812
MC 0	79%	14.91	5.934	16.64	5.322	22.57	3.934	5.970	−24.418
MC 1P	39%	14.74	6.002	16.46	5.380	22.62	3.927	4.760	−18.137
MC 10P	42%	14.71	6.014	16.35	5.416	22.60	3.930	4.770	−19.353
MC 25P	69%	14.75	6	16.64	5.323	22.64	3.924	4.890	−13.330

**Table 2:** Average diameter, polydispersity index, and Zeta potential determined by dynamic light scattering (DLS).

Concentration	5.7 × 10^−4^% *w*/*v*			5.7 × 10^−5^% *w*/*v*			
Sample	*D*_m_, nm	PdI	P1...n, nm	*D*_m_, nm	PdI	P1...n, nm	Zeta, mV
K_M1P	2199	1.000	P1 = 276	568	0.453	P1 = 436	−10.4
K_M10P	1617	0.154	P1 = 1873	632	0.480	P1 = 558 P2 = 54	−10.2
K_M25P	1117	0.551	P1 = 677	1183	0.684	P1 = 1366 P2 = 170	−13.1
MC_1P	906	0.236	P1 = 848	419	0.392	P1 = 422 P2 = 74	−28.6
MC_10P	830	0.530	P1 = 644 P2 = 74	597	0.815	P1 = 604 P2 = 95	−32.5
MC_25P	643	0.406	P1 = 477	674	0.419	P1 = 621 P2 = 66	−20.8

**Table 3:** Values of the onset degradation temperature (*T*_on_), the temperature at the maximum degradation rate (*T*_d_) and residue for Kombucha-derived cellulose samples.

Sample	*T*_on_	*T*_d_	Residue at 700 °C
K 0	298.7	346.6	26.5
K 4M	253.9	273.9/328.3	28.8
K AT	329.4	363.5	11.9
K CM	282.5	338.0	13.7
MC 0	320.6	339.3	4.0
